# Molecular Detection and Multilocus Genotyping of *Giardia duodenalis* in Pigs in Fujian Province, Southeastern China

**DOI:** 10.3390/ani12223148

**Published:** 2022-11-14

**Authors:** Fu-Rong Zhao, Ning Zhang, Wen-Yuan Miao, Ran Wu, Lin-Lin Cui, Cui-Qin Huang, Dong-Hui Zhou

**Affiliations:** 1 Fujian Key Laboratory on Conservation and Sustainable Utilization of Marine Biodiversity, Fuzhou Institute of Oceanography, Minjiang University, Fuzhou 350108, China; 2 Key Laboratory of Fujian-Taiwan Animal Pathogen Biology, College of Animal Sciences (College of Bee Science), Fujian Agriculture and Forestry University, Fuzhou 350002, China; 3 Fujian Provincial Key Laboratory for the Prevention & Control of Animal Infectious Diseases & Biotechnology, Longyan University, Longyan 364012, China; 4 Engineering Research Center for the Prevention and Control of Animal Original Zoonosis, Fujian Province University & College of Life Science, Longyan 364012, China

**Keywords:** *Giardia duodenalis*, pigs, prevalence, genotype, multilocus sequence typing, southeastern China

## Abstract

**Simple Summary:**

Giardia duodenalis is a world-widely distribution intestinal protozoan parasite that can infect human and a broad range of mammals. It can cause a number of clinical symptoms including diarrhoea, abdominal pain, vomiting, severe dehydration and anemia etc. in animals and humans. Eight genetic assemblages (A to H) have been identified in G. duodenalis based on molecular analysis. In this study, the infection rates and genotypes of G. duodenalis in swine are investigated in Fujian province, southeastern China. G. duodenalis assemblages E was found in pigs in the present study. These results provide epidemiological data for giardiasis prevention, control and public health.

**Abstract:**

*Giardia duodenalis*, an intestinal parasite, is widely distributed in humans and various animals, such as pigs, cattle and cats. The clinical symptoms of giardiasis are characterized as including abdominal pain, acute or chronic diarrhea, and bloating and weight loss in humans and animals, leading to public and veterinary health problems worldwide. However, the prevalence and genotypes of *G. duodenalis* in pigs in Fujian Province, southeastern China, have not been reported. In the present study, 725 fecal samples were collected from six cities (Fuqing, Putian, Nanping, Longyan, Sanming, Zhangzhou) in Fujian Province and analyzed for *G. duodenalis* prevalence and genotypes using nested PCR targeting the beta-giardin (*bg*), glutamate dehydrogenase (*gdh*) and triosephosphate isomerase (*tpi*) genes. The results shown that total occurrence rate of *G. duodenalis* was 26.9% (195/725) in pigs, with significant differences in the prevalence among different regions (*χ*^2^ = 86.508, *p* < 0.05) and groups (*χ*^2^ = 12.748, *p* < 0.05). 195, 11 and 6 samples were detected at the *bg*, *tpi* and *gdh* loci, respectively. Each one belonged to a subtype of assemblage E and was analyzed using sequences obtained in this study. Based on phylogenetic analyses of sequences from the three genetic loci, only one MLG E1 was found. The results indicated that pigs may present a potential zoonotic risk of spreading *G. duodenalis* infection from animals to humans in this area. The findings of the present study also provide basic data for the prevention and control of *G. duodenalis* infection in pigs and humans in China.

## 1. Introduction

*Giardia duodenalis* (syn, *G. intestinalis* or *G. lambila*) is an intestinal protozoan parasite distributed worldwide and transmitted through the fecal-oral route [1]. Humans and animals are infected by ingesting *G. duodenalis* cyst-contaminated water and food or by direct touch with infected animals, which causes abdominal pain, acute or chronic diarrhea, and bloating and weight loss [2,3]. Acute giardiasis develops after an incubation period of 1 to 14 days (average of 7 days) and usually lasts 1 to 3 weeks. Repeated samplings are usually required on different days during diagnosis because Giardia cysts and trophozoites are not always present in the feces. So far, no effective vaccines have been able to prevent and control giardiasis. There are some antimicrobial drugs for treatment of giardiasis, such as metronidazole, nitazoxanide, paromomycin, tinidazole, quinacrine and furazolidone. However, these drugs can cause side effects (e.g., metallic taste) and drug resistance in many poor patients [1,4]. Some studies have reported a high prevalence and disease burden of *G. duodenalis* infection in animals, such as pigs, cattle, dogs, cats and wildlife [5,6,7]. Thus, *G. duodenalis* has a significant health impact on both humans and animals.

To date, eight assemblages (A–H) have been identified in *G. duodenalis* based on genetic analysis. Among them, assemblages A and B are found in both humans and a variety of mammals, and the remaining six assemblages are strongly host-specific [8,9]. It is commonly believed that humans are infected only with assemblages A and B, but the sporadic infection by assemblages C, D, E and F have also been isolated from humans [10,11,12,13]. At present, there are prevalence reports and genotype analyses of *G. duodenalis* infection in pigs all over the world. In the published studies, assemblages A, B and E were identified in pigs, with assemblage E being the predominant genotype [14,15,16,17].

Due to the inadequate genetic characterization of *G. duodenalis* using only a single gene, and even sometimes a limited sensitivity, genotyping of *G. duodenalis* is most commonly achieved by the amplification of several target genes, such as the small subunit of ribosomal RNA (*SSU rRNA*), beta-giardin (*bg*), triose phosphate isomerase (*tpi*) and glutamate dehydrogenase (*gdh*) [1,8]. In recent years, multilocus genotyping (MLG) based on the three genes (*bg*, *tpi* and *gdh*) was developed and has been applied to provide more genetic information and contribute to understanding possible zoonotic transmission linkages [18,19]. However, limited data on *G. duodenalis* infection in pigs in China is available. Thus, the current study was intended to characterize *G. duodenalis* in pigs in Fujian Province and to assess the public health potential of *G. duodenalis* in pigs.

## 2. Materials and Methods

### 2.1. Specimen Collection

**Figure 1:**
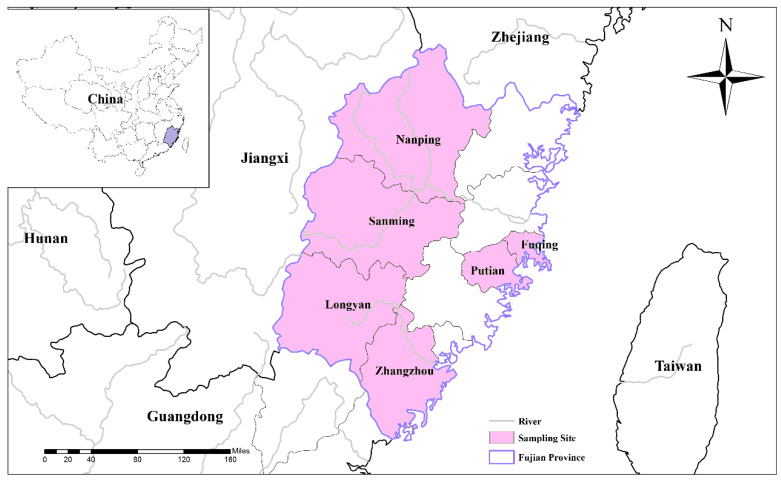
Geographic distribution of sampling sites in Fujian Province, southeastern China.

In total, 725 fresh fecal samples were collected from six regions (121 from Fuqing, 146 from Putian, 139 from Nanping, 120 from Longyan, 118 from Sanming and 81 from Zhangzhou) in Fujian Province (Location: 24′ N to 28′ N and 116′ E to 121′ E), southeastern China (Figure 1). These samples were collected from industry farms with more than 500 breeding sows. The distribution of samples by type was as follows: 111 samples from suckling pigs, 127 samples from weaned pigs, 104 samples from nursery pigs, 298 samples from sows, 28 samples from boars and 57 samples from fattening pigs. All the fecal samples were placed in clean plastic bags and preserved in 2.5% potassium dichromate (4 °C) prior to DNA extraction.

### 2.2. DNA Extraction and Nested PCR Amplification

Approximately 200 mg of feces per sample was placed in a sterile centrifuge tube, and the potassium dichromate solution was washed three times with distilled water. Genomic DNA was extracted using the commercial *E.Z.N.A*^®^ Stool DNA kit (Omega Biotek Inc., Norcross, GA, USA) according to the manufacturer’s protocol. *G. duodenalis* in fecal specimens was determined using nested PCR analysis of the beta-giardin (*bg*) gene. The primers and amplification used in this study have been previously described [1,7]. The final amplification products were subjected to electrophoresis in 1% agarose gel stained with Gold View^TM^ (Solar bio Co., Ltd., Beijing, China) and were visualized under UV light.

### 2.3. Sequence Analysis

*G. duodenalis* positive secondary PCR products from the *bg*, *tpi* and *gdh* genes were sequenced bidirectionally with secondary PCR primers using an ABI PRISM^TM^ 3730 XL DNA Analyzer (Applied Biosystems, Foster City, CA, USA) at the Sune Biotech Co., Ltd. (Fuzhou, China). Then, the obtained DNA sequences were aligned with references downloaded from the National Center for Biotechnology Information (NCBI) website (http://www.ncbi.nlm.nih.gov/BLAST (accessed on 16 August 2022)) using the software Clustal X v.2.1 (http://www.clustal.org/ (accessed on 16 August 2022)) to identify *G. duodenalis* assemblages/subtypes.

### 2.4. Phylogenetic Analysis

MEGA X version 10.1.7 (http://www.megasoftware.net (accessed on 16 August 2022)) software was used to perform phylogenetic analyses using the neighbor-joining method with the Kimura 2-parameter model. Bootstrap analyses with 1000 replicates were used to assess the robustness of cluster formation.

### 2.5. Statistical Analysis

Differences in infection rate between husbandry parameters, including groups and locations, were compared with the chi-square test (*χ*^2^) using the software SPSS 22.0 (IBM Corp., New York, NY, USA). Values were considered to be statistically significant when *p* < 0.05.

## 3. Results

From a total of 725 fecal specimens in this study, 195 (26.9%) fecal samples tested positive for *G. duodenalis* based on the beta-giardin (*bg*) gene. The highest infection rate was seen in specimens from Sanming (49.2%, 58/118), followed by Nanping (36.7%, 51/139), Fuqing (36.4%, 44/121), Zhangzhou (22.2%, 18/81), Longyan (10.8%, 13/120) and Putian (7.5%, 11/146), and was statistically significant (*χ*^2^ = 86.508, *p* < 0.05). Among the six groups of pigs, suckling pigs (35.1%, 39/111) had the highest infection rate, followed by weaned pigs (29.1%, 37/127), boars (28.8%, 8/28), sows (27.2%, 81/298), nursery pigs (23.1%, 24/104) and fattening pigs (10.5%, 6/57), and the difference was statistically significant (*χ*^2^ = 12.748, *p* < 0.05) (Table 1).

**Figure 2:**
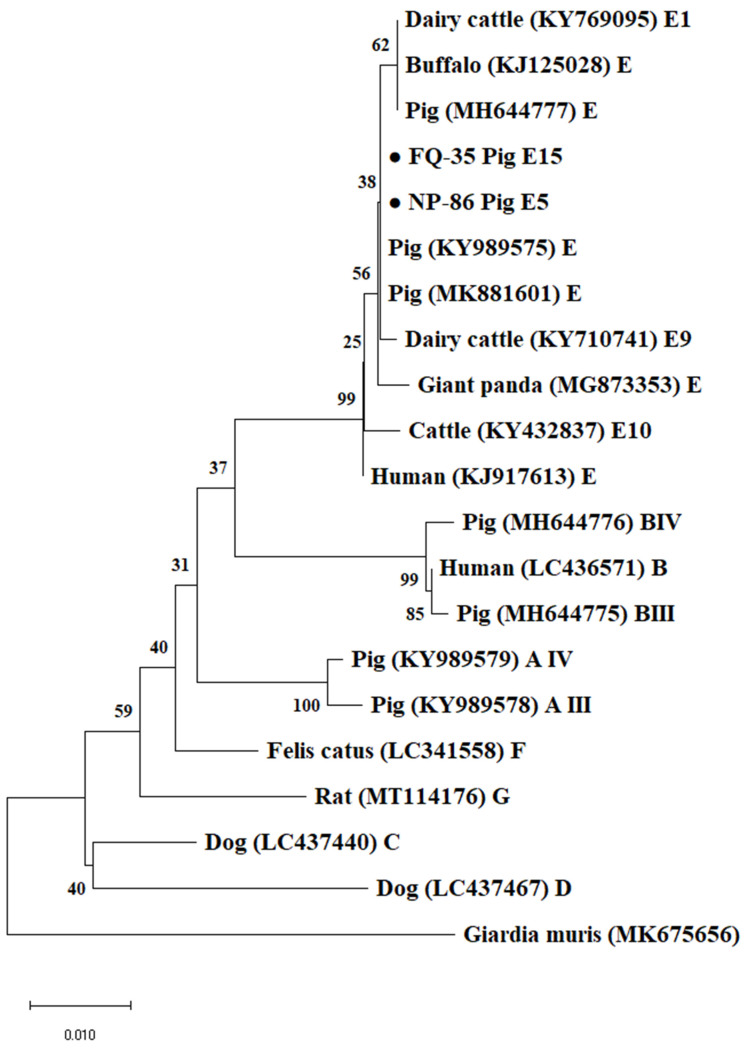
Phylogenetic relationships of *Giardia duodenalis* isolates obtained using the neighbor-joining analysis of the beta-giardin (*bg*) nucleotide sequences. Bootstrap values >50% from 1000 replicates are shown as nodes. *Giardia muris* was used as outgroup. Filled black circles represent *bg* sequences generated in this study. The scale bar indicates 0.01 nucleotide substitution/site.

**Figure 3:**
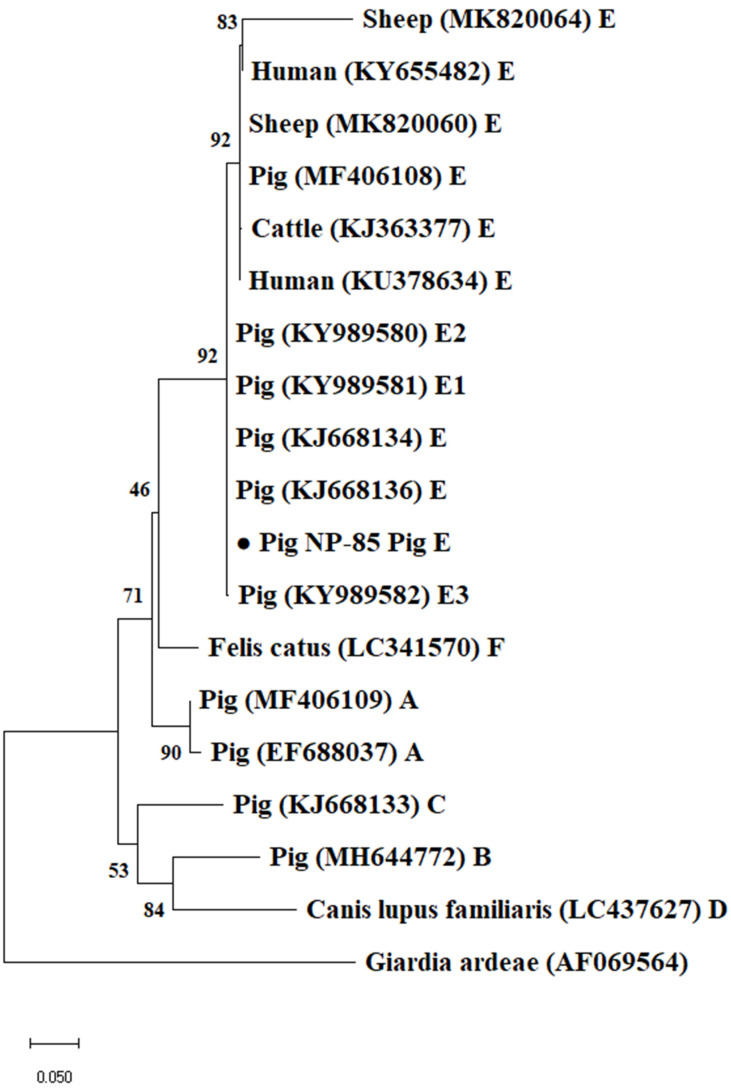
Phylogenetic relationships of *Giardia duodenalis* isolates obtained using the neighbor-joining analysis of the glutamate dehydrogenase (*gdh*) nucleotide sequences. Bootstrap values >50% from 1000 replicates are shown as nodes. *Giardia ardeae* was used as outgroup. Filled black circles represent *bg* sequences generated in this study. The scale bar indicates 0.2 nucleotide substitution/site.

**Figure 4:**
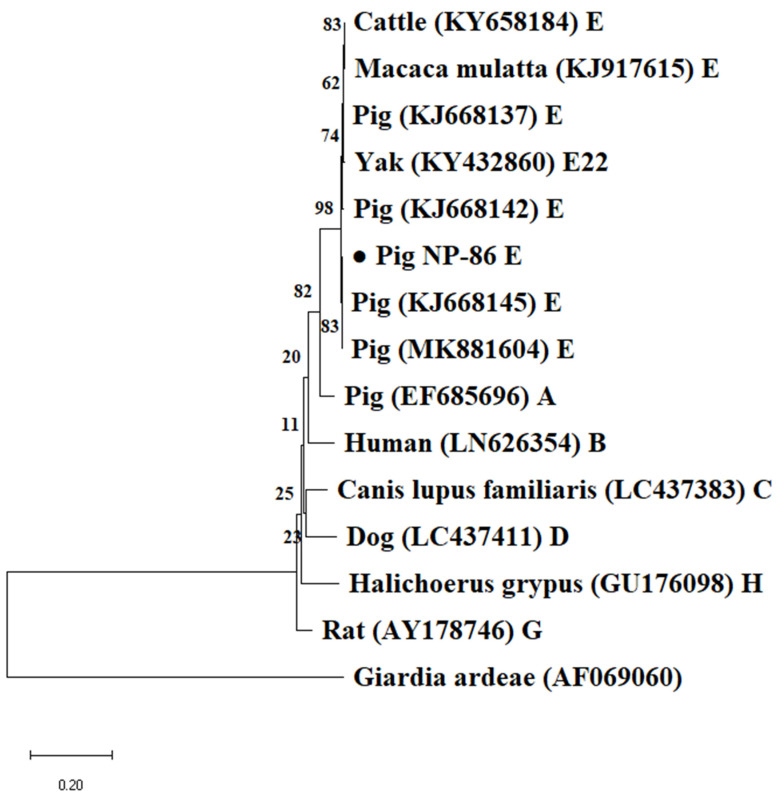
Phylogenetic relationships of *Giardia duodenalis* isolates obtained using the neighbor-joining analysis of the triose phosphate isomerase (*tpi*) nucleotide sequences. Bootstrap values >50% from 1000 replicates are shown as nodes. *Giardia ardeae* is used as outgroup. Filled black circles represent *bg* sequences generated in this study. The scale bar indicates 0.05 nucleotide substitution/site.

Based on the *bg* gene locus, all 195 *G. duodenalis* positive samples belonged to assemblage E, among which subtypes E5 and E15 were found, and also showed 100% nucleotide identity with a cattle-derived sequence (GenBank accession number: MH158458) and a Tibetan sheep-derived sequence (GenBank accession number: KY633473) respectively. In total, 11 and 6 *Giardia*-positive samples belonging to assemblage E15 were identified at the *tpi* and *gdh* gene loci, respectively, and showed 100% nucleotide identity with a pig-derived sequence (GenBank accession number: KJ668136) and another pig-derived sequence (GenBank accession number: KJ668145), respectively. Unfortunately, attempts to access data at the *tpi* and *gdh* gene loci failed in all assemblage E5 samples. Phylogenetic analyses show that all sequences generated in this study at the *gdh*, *bg* and *tpi* loci were grouped in well-defined clusters with suitable references and matched previously published sequences retrieved from GenBank (Figure 2, Figure 3 and Figure 4).

Six samples were amplified at three loci at the same time and only one MLG E1 was formed (Table 2).

## 4. Discussion

*G. duodenalis* infection in pigs has been reported worldwide. In this study, the total infection rate of *G. duodenalis* was 26.9% (195/725), which was close to the infection rate detected using PCR based on the *bg*, *tpi* and *gdh* loci and using ELISA in Shanghai (26.88%) [20] and Nigeria (25.4%) [21] in 2019. The infection rate in the present study detected using PCR based on the SSU rRNA and *bg* loci was lower than the following that were reported: 31.1% in Australia in 2009 [22], 66.4% in Canada in 2011 [23] and 57.1% in the United Kingdom in 2014 [24]. However, the infection rate in the present study detected using microscopy and PCR based on the SSU rRNA, *bg*, *tpi* and *gdh* loci was higher than most reports on G. duodenalis: 17.4% in Denmark in 2006 [25], 7.4% in the United States in 1994 [26], 9.5% in Poland in 2015 [27], 8% in Shaanxi in 2017 [15], 14.8% in Korea in 2020 [28], 4.26% in Taiwan in 2021 [29] and 0.97% in Hubei in 2022 [30]. Many factors, such as testing methods, sample size, sampling time and geographic location, may provide reasons for the differences in *G. duodenalis* prevalence.

Infection rates were statistically significant among different groups (*χ*^2^ = 86.508, *p* < 0.05), with the highest prevalence in suckling pigs and the lowest in fattening pigs, which were similar to those of Australia and Denmark [22,31]. However, the study on the infection of *G. duodenalis* in Shaanxi Province showed that the infection rate of sows was the highest (10.5%) [15], which was consistent with the result in Lusaka, Zambia (53.3%) [32]. Multiple factors, such as loci amplified, climate, age structure and sample size, may lead to various infection rates.

The alignment of nucleotide sequences obtained in this investigation showed only one genotype of assemblage E in Fujian Province. The previous report shows that ungulates, including swine, are easily infected by assemblage E, which can also infect humans [10]. This indicates that assemblage E presents a risk for humans and animals. Study results about *G. duodenalis* in Shaanxi Province found that the main zoonotic genotype was assemblage A (20%) [15]. Assemblage E and A mixed infections were observed in Denmark [25]. The distribution of assemblages in different regions is slightly different, so further studies are needed on the epidemiology and genotypic transmission mechanism of human and swine *G. duodenalis.*

In total, six samples were amplified at three loci at the same time. Only one MLG E1 was formed. In Shaanxi Province, eight samples were amplified to form four MLGs at the three loci of *bg*, *tpi* and *gdh*. In addition, coinfection with assemblage A and assemblage E was identified in one fattening pig [15]. The results showed that polymorphism in *G. duodenalis* was different among regions. However, more data are needed on the specific mechanism due to few reports on the MLG typing of *G. duodenalis* in pigs.

*G. duodenalis* induces a strongly adaptive immune response in both humans and animals. After infection, a large number of parasitic-specific IgA are produced, and the CD4^+^ T cell response is helpful for the production of IgA and the control of infection [33]. Piglets do not become fully immune until they are about 4 weeks old after birth, which means they are less able to respond to the source of infection during the first month [34]. This is consistent with the highest rate of infection in suckling pigs in our survey. Despite the global spread of *G. duodenalis* infection, there is, unfortunately, currently no protective vaccine available for human use, and drug treatment regimens have had varying effects [35]. As an omnivorous and monogastric species, pigs are very similar to humans in terms of anatomical structure and immune system function. Studies have reported that the similarity between the immune systems of pigs and humans is more than 80% [36]. Therefore, pigs have many advantages as animal models of human diseases.

## 5. Conclusions

In conclusion, the total infection rate of *G. duodenalis* was 26.9% (195/725) in pigs in Fujian Province, southeastern China, and only one MLG was formed. To the best of our knowledge, this is the first report of *G. duodenalis* infection in pigs in Fujian Province. Considering that pigs are necessary economic animals in China, a better understanding of *G. duodenalis* in pigs will help to develop more targeted prevention and control measures, which have essential implications for the control of *G. duodenalis* infection in pigs and humans.

## Figures and Tables

**Table 1:** Prevalence of *G. duodenalis* infection in pigs in Fujian Province.

Variable	Category	No. Isolates	No. Positive (%, 95% CI)	Assemblage (Number)	*p*-Value
Group	Suckling pigs	111	39 (35.1%, 26.9–44.4%)	E (39)	<0.05
	Weaned pigs	127	37 (29.1%, 21.9–37.5%)	E (37)	
	Nursery pigs	104	24 (23.1%, 16.0–32.1%)	E (24)	
	Fattening pigs	57	6 (10.5%, 4.9–21.1%)	E (6)	
	Sows	298	81 (27.2%, 22.4–32.5%)	E (81)	
	Boars	28	8 (28.6%, 15.3–47.1%)	E (8)	
Location	Fuqing City	121	44 (36.4%, 28.3–45.2%)	E (44)	<0.05
	Nanping City	139	51 (36.7%, 29.1–44.9%)	E (51)	
	Longyan City	120	13 (10.8%, 6.4–17.6%)	E (13)	
	Putian City	146	11 (7.5%, 4.2–12.9%)	E (11)	
	Sanming City	118	58 (49.2%, 40.3–58.1%)	E (58)	
	Zhangzhou City	81	18 (22.2%, 14.5–32.4%)	E (18)	
	Total	725	195 (26.9%, 23.8–30.2%)	E (195)	

**Table 2:** Multilocus characterization of *G. duodenalis* isolates based on *bg*, *tpi* and *gdh* genes.

Isolate	Genotype or Subtype			MLG Type
	*bg*	*tpi*	*gdh*	
NP-68	E15	E1	E1	MLG E1
NP-69	E15	E1	E1	MLG E1
NP-71	E15	E1	E1	MLG E1
NP-73	E15	E1	E1	MLG E1
NP-79	E15	E1	E1	MLG E1
NP-81	E15	E1	-	-
NP-85	E15	E1	-	-
NP-86	E15	E1	E1	MLG E1
NP-70	E15	E1	-	-
NP-78	E15	E1	-	-
PT-YF3	E15	E1	-	-

## Data Availability

Not applicable.
